# Association between obesity and mental health problems among Spanish children aged 9 and 12 years: the ELOIN study

**DOI:** 10.1186/s12889-026-26349-w

**Published:** 2026-02-07

**Authors:** Honorato Ortiz-Marrón, Gloria Cabañas Pujadas, Ana Gandarillas Grande, María Victoria Martínez Rubio, Eva María Cabrero López, María Teresa Morales San José, Águeda Quadrado Mercadal, Amelia Astray San Martín, María Isabel Careaga González, Marta González Alcón, Isabel Férriz Vidal, Iñaki Galán

**Affiliations:** 1 Department of Non-Communicable Disease Surveillance, General Directorate of Public Health, Community of Madrid, Ministry of Health, Madrid, Spain; 2 Department of Health, Food Safety and Public Health, Ministry of Health, Madrid, Spain; 3 Paediatrician at Los Fresnos Health Centre, Community of Madrid, Ministry of Health, Torrejón de Ardoz, Spain; 4 Paediatrician at Adelfas Health Centre, Community of Madrid, Ministry of Health, Madrid, Spain; 5 Paediatrician at La Paz Rivas Health Centre, Community of Madrid, Ministry of Health, Rivas-Vaciamadrid, Spain; 6 Paediatrician at Navas del Rey Health Centre, Community of Madrid, Ministry of Health, Navas del Rey, Spain; 7 Paediatrician at Guadalix de la Sierra Health Centre, Community of Madrid, Ministry of Health, Guadalix de la Sierra, Spain; 8 Primary care nurse at Doctor Cirajas Health Centre, Community of Madrid, Ministry of Health, Madrid, Spain; 9 Paediatrician at Lavapies Health Centre, Community of Madrid, Ministry of Health, Madrid, Spain; 10 Paediatrician at Valdelasfuentes Health Centre, Community of Madrid, Ministry of Health, Alcobendas, Spain; 11 National Centre for Epidemiology, Institute of Health Carlos III, Madrid, Spain; 12 Department of Preventive Medicine and Public Health, Autonomous University of Madrid, Madrid, Spain; 13 Department of Non-Communicable Disease Surveillance, General Directorate of Public Health, Community of Madrid, C/López de Hoyos, no. 35, 1 st floor, Madrid, 28002 Spain

**Keywords:** Childhood obesity, Emotional and behavioural problems, SDQ, Psychological problems

## Abstract

**Background:**

Obesity is an important determinant of mental health in the child and adolescent population. In this study, the association between obesity and the development of psychological problems in Spanish children aged 9 and 12 years was examined.

**Methods:**

In this prospective study, 2,473 9-year-old children and 2,119 12-year-olds who participated in the Longitudinal Study of Childhood Obesity (ELOIN) were evaluated. The standardized BMI was calculated from weight and height, and weight status was classified according to the 2007 World Health Organization criteria. Mental health difficulties were assessed using the Strengths and Difficulties Questionnaire (SDQ), which was completed by the parents. BMI and SDQ scores were determined repeatedly at 9 and 12 years of age. Using generalized estimating equations, β coefficients were calculated when analysing the SDQ scores, and odds ratios (ORs) were calculated to estimate mental health risk using Goodman´s British cut-off points.

**Results:**

Compared with participants without obesity, those who had obesity had a higher total score on the SDQ (β coefficient: 1.12 (95% CI: 0.59; 1.64)) and an increased risk of mental health risk (OR: 1.36 (95% CI: 1.09; 1.69)). ORs of 1.48 (95% CI: 1.21; 1.81) for emotional problems, 1.40 (95% CI: 1.15; 1.70) for conduct problems, and 2.25 (95% CI: 1.83; 2.77) for problems with peers were estimated. This association of weight status with mental health difficulties was moderately greater in girls than in boys, although the difference was not statistically significant.

**Conclusions:**

Childhood obesity was associated with mental health difficulties, especially emotional symptoms, behavioural problems and relationships with peers. Identifying and responding to these psychological problems should also be an objective in public health policies and procedures for controlling obesity in children.

**Supplementary Information:**

The online version contains supplementary material available at 10.1186/s12889-026-26349-w.

## Background

The incidence of childhood obesity has increased rapidly worldwide in recent decades [1]. Currently, in low- and middle-income countries, the prevalence of obesity continues to increase, while in many Western countries, trends have stabilized, although the prevalence of obesity remains high [1]. According to the Childhood Obesity Surveillance Initiative (COSI) of the World Health Organization (WHO) and the ALADINO study, Spain is among the five countries with the highest rates of obesity in the European region, with a prevalence of excess weight of 41.3% in boys and 39.7% in girls and a prevalence of obesity of 19.4% and 15.0%, respectively [2].

On the other hand, mental health problems also cause a large global burden of disease in the child and adolescent population. The WHO estimates that one in seven adolescents aged 10 to 19 years suffers from a mental disorder [3]. A recent meta-analysis reported that the prevalence of mental problems in childhood in 11 Western countries between 2003 and 2020 was 12.7%, with the most prominent mental problems being anxiety and depression disorders, attention-deficit/hyperactivity disorder, behaviour disorders, and substance use disorder [4].

Obesity during childhood, which is likely to continue into adulthood [5], is associated with cardiometabolic and psychosocial comorbidities and increased premature mortality [6]. Obesity can have consequences not only for physical health but also for mental health and quality of life in children and adolescents [7], including depression and anxiety [8], low self-esteem, lower quality of life [9, 10], and other emotional and behavioural problems [11–13]. In fact, the findings of a recent study suggested that treating obesity in children can reduce emotional and behavioural problems [14]. Children and adolescents with excess weight also report greater sleep problems [15] and more frequently suffer from pain and lumbago [16]. On the other hand, greater problems with relationships with peers and internalizing and externalizing behaviours have also been described in children and adolescents with obesity [17, 18]. In addition, other studies have shown that obesity is associated with psychosocial problems, such as stigma, exclusion, teasing, and bullying from peers [19].

However, despite the well-established relationship between obesity and the increased risk of psychological comorbidities in individuals living with obesity [20], scientific evidence highlighting, for example, the different impacts that obesity can have on boys and girls in childhood and preadolescence is limited [21]. Therefore, the aim of this study was to estimate the association between obesity and mental health problems, measured by the Strengths and Difficulties Questionnaire (SDQ), which addresses social, emotional and behavioural dimensions, in a sample of Spanish children aged 9 and 12 years and to evaluate differences according to sex.

## Methods

### Type of study and population

In this prospective study, repeated measurements were obtained from children aged 9 and 12 years. The data come from the Longitudinal Study of Childhood Obesity (ELOIN), a population-based prospective study consisting of a representative baseline cohort of the 4-year-old population of the Community of Madrid (7 million inhabitants); ELOIN methodology has been previously published [22]. The ELOIN consists of standardized anthropometric measurements and a telephone interview completed by the children’s parents at 4, 6, 9 and 12 years of age.

A total of 2,844 children were included in the study. 2,473 participated in the measurement at age 9, of whom 1,746 also participated at age 12. The measurement at age 12 included 373 children who were examined only at that age. Therefore, the number of participants at age 12 was 2,119.

### Anthropometric measurement and weight status

The physical examinations were performed by paediatricians and nurses from the Sentinel Network of the 31 primary care health centres participating in the study [23]. Standardized measurements of weight and height were taken. Weight measurements were made using a digital scale, and height measurements were collected with a telescopic height rod. BMI (kg/m2) was estimated based on age (months) and sex. Weight status was categorized with the 2007 WHO reference tables. Based on BMI z scores, underweight was defined as a zBMI ≤ 2 standard deviations (SDs) below the mean, normal weight as a zBMI ≥ 2 SDs and ≤ 1 SD of the mean, overweight as a zBMI > 1 SD and ≤ 2 SD above the mean, and obesity as a zBMI > 2 SD above the mean [24].

### Behavioural strengths and difficulties

Emotional, behavioural and relationship problems were assessed using the Strengths and Difficulties Questionnaire (SDQ). It is a screening tool that measures positive and negative psychological attributes in children and adolescents [25, 26]. In this study, the version for parents was used, for which adequate validity and reliability in Spanish children have been shown [27]. The SDQ consists of 5 subscales/dimensions with 25 questions: Four of these subscales measure negative aspects of mental health difficulties, such as conduct problems (e.g. often has temper tantrums or hot tempers), emotional problems (e.g. often complains of headaches), hyperactivity (e.g. constantly fidgeting or squirming), and problems with peers (e.g. rather solitary, tends to play alone). One subscale measures prosocial behaviour, which includes positive behaviours (e.g. considerate of other people´s feeling). Each question consists of three possible responses scored from 0 to 2 (“Not true” = 0, “Somewhat true” = 1 and “Absolutely true” = 2), with a maximum score of 10 for each subscale. The sum of the scores on the 4 subscales related to negative aspects is the Total Difficulty Scale (TDS-SDQ) score, which ranges from 0 to 40. High scores indicate greater difficulty, except for the prosocial scale, in which lower scores indicate greater difficulty. For the risk cut-off points of the TDS-SDQ and the subscales, the criteria proposed by Goodman in the British population were used [28, 29]. A total value ≥ 14 is considered borderline/abnormal (borderline: ranking from 14 to 16 and abnormal: ranking from 17 to 40) and indicative of a possible mental health problem. In the dimensions of the SDQ, the following borderline/abnormal cut-off points were considered: ≥4 for emotional problems, ≥ 3 for behavioural problems, ≥ 6 for hyperactivity, and ≥ 3 for problems with peers. For prosocial behaviour, the cut-off point was ≤ 5.

### Covariates

The sociodemographic variables collected through telephone interviews with the parents were sex, age, and family purchasing power. Family purchasing power was estimated using the score on the *Family Affluence Scale* (FAS-II), which is a global indicator of family socioeconomic status, classified as low (0–3 points), medium (4–5 points), or high (6–9 points) [30].

Other covariates were the quality of the diet and physical activity. Diet quality was evaluated according *to the Mediterranean Diet Quality Index* (Med-DQI) [31] using a semiquantitative food consumption frequency questionnaire that records the frequency of consumption (daily, weekly, monthly, or yearly) of 145 foods; the index score ranges from 0 to 14 points, and a higher score is indicative of a poorer quality of the diet. Physical activity was determined from the validated *Physical Activity Questionnaire-Children* (PAQ–C), for which scores range from 1 (little physical activity) to 5 (very much physical activity) [32].

### Statistical analysis

Descriptive statistics are expressed as percentages and means. Analysis of variance (ANOVA) was used to estimate the differences in the means of the groups analysed (age, sex, weight status, etc.), and Pearson’s chi-square test was used to determine the differences in categorical variables between groups. The analyses were performed separately for boys and girls because previous research has suggested that the distribution of SDQ scores differs according to sex [33].

The association between obesity and mental health problems was analysed using generalized estimating equation (GEE) models that take into account the correlation between measurements at 9 and 12 years of age. To assess the association between obesity and the TDS-SDQ score and scores on its dimensions, β coefficients were estimated from GEE linear regression (GEE linear models), while to analyse the relationship with mental health risk (TDS-SDQ score and scores on its dimensions) in a categorized manner, odds ratios (ORs) were estimated using GEE logistic models. All GEE models were adjusted for sex, age, family purchasing power, quality of diet, and physical activity. To assess the possible modification of the effect according to sex, the statistical significance of the interaction was evaluated (p-value < 0.05). In addition, to evaluate whether the association between obesity and mental health changed between ages 9 and 12, we included an interaction term between these variables.

Finally, two sensitivity analyses were carried out: (1) one analysis excluding 92 underweight children, and (2) one analysis in which the weight status was stratified into normal weight, overweight and obese.

Confidence intervals (CIs) were established at 95% for all estimators. The level of statistical significance was set at 0.05.

All analyses were performed with Stata version 18 (StataCorp, College Station, Texas, USA).

#### Ethical implications

 The study protocol was approved by the Clinical Research Ethics Committee (CEIC) of the Ramón y Cajal University Hospital in Madrid. The parents/guardians of the minors provided informed consent in writing. The present study was carried out in accordance with the guidelines of the Declaration of Helsinki, and all methods were carried out following the relevant guidelines and regulations.

## Results

Table 1 shows the characteristics of the sample at 9 and 12 years of age according to sex and obesity status. A total of 51.2% and 51.6% of the participants aged 9 and 12 years, respectively, were girls. A total of 15.3% and 11.7% of the participants had obesity at 9 and 12 years, respectively. The prevalence of obesity was greater in boys than in girls both at 9 years (17.8% vs. 12.9%) and at 12 years (15.3% vs. 8.3%). A total of 17.0% and 19.9% of the children aged 9 and 12 years with obesity lived in low-income households, while 10.6% and 11% of the children without obesity lived in low-income households, respectively (*p* < 0.01).

**Table 1:** Characteristics of the sample at 9 and 12 years of age according to weight status

Children 9 years of age	Total (*n* = 2473)		Boys (*n* = 1207)		Girls (*n* = 1266)	
	No obesity	Obesity ^a^	No obesity	Obesity ^a^	No obesity	Obesity ^a^
Total, n (%)	2095 (84.7)	378 (15.3)	992 (82.2)	215 (17.8)	1103 (87.1)	163 (12.9)*
Age (months), mean (SD)	111.3 (4.9)	110.8 (4.0)	111.1 (4.8)	110.8 (3.8)	111.5 (5.1)	110.8 (4.2)
Household purchasing power ^b †^ (%)						
Low	213 (10.6)	61 (17.0)**	108 (11.3)	28 (13.7)*	105 (10.0)	33 (21.3)**
Medium	436 (21.8)	106 (29.5)	196 (20.6)	60 (29.4)	240 (22.8)	46 (29.7)
High	1355 (67.6)	192 (53.5)	648 (68.1)	116 (56.9)	707 (67.2)	76 (49.0)
Physical activity ^c †^, mean (SD)	3.1 (0.6)	2.9 (0.6)*	3.2 (0.6)	3.0 (0.6)**	3.0 (0.6)	2.9 (0.7)*
Diet quality score ^d †^, mean (SD)	6.4 (1.6)	6.3 (1.7)	6.4 (1.6)	6.4 (1.7)	6.3 (1.6)	6.1 (1.7)
Total Difficulties Score (TDS-SDQ) ^e^, mean (SD)	8.7 (5.2)	10.5 (5.8)**	9.0 (5.3)	10.8 (5.9)**	8.4 (5.1)	10.1 (5.8)**
Emotional symptoms, mean (SD)	2.1 (1.9)	2.5 (2,3) **	2.0 (1.8)	2.3 (1.9)*	2.1 (1.9)	2.7 (2.2)**
Conduct problems, mean (SD)	1.7 (1.5)	2.1 (1.6)**	1.8 (1.5)	2.2 (1.7)*	1.6 (1.4)	1.9 (1.4)*
Hyperactivity, mean (SD)	3.9 (2.6)	4.1 (2.5)	4.1 (2.6)	4.4 (2.6)	3.6 (2.5)	3.8 (2.3)
Peer relationship problems, mean (SD)	1.0 (1.4)	1.8 (1.8)**	1.0 (1.3)	1.9 (1.9)**	1.0 (1.4)	1.7 (1.7)**
Prosocial behaviour ^f^, mean (SD)	8.8 (1.4)	8.7 (1.4)	8.6 (1.5)	8.5 (1.5)	9.0 (1.3)	8.9 (1.2)

Children 12 years of age	Total (*n* = 2119)		Boys (*n* = 1025)		Girls (*n* = 1094)	
	No obesity	Obesity ^a^	No obesity	Obesity ^a^	No obesity	Obesity ^a^
Total, n (%)	1872 (88.3)	247 (11.7)	868 (84.7)	157 (15.3)	1004 (91.8)	90 (8.3)**
Age (months), mean (SD)	146.9 (4.1)	146.7 (4.4)	146.9 (4.2)	147.1 (4.2)	146.9 (4.0)	146.2 (4.7)
Household purchasing power ^b †^ (%)						
Low	205 (11.0)	49 (19.9)**	100 (11.6)	30 (19.2)**	105 (10.5)	19 (21.1)*
Medium	514 (27.5)	85 (34.6)	241 (27.8)	59 (37.8)	273 (27.2)	26 (28.9)
High	1150 (61.5)	112 (45.5)	525 (60.6)	67 (43.0)	625 (62.3)	45 (50.0)
Physical activity ^c †^, mean (SD)	2.6 (0.6)	2.6 (0.6)	2.7 (0.6)	2.8 (0.6)*	2.6 (0.6)	2.5 (0.6)
Diet quality score ^d †^, mean (SD)	7.0 (1.7)	6.9 (1.7)	7.0 (1.7)	7.0 (1.7)	6.9 (1.6)	6.6 (1.6)
Total Difficulties Score (TDS-SDQ) ^e^, mean (SD)	8.0 (5.5)	9.3 (5.9)**	8.6 (5.7)	9.2 (5.9)	7.7 (5.4)	9.5 (6.0)*
Emotional symptoms, mean (SD)	2.2 (2.1)	2.6 (2.4)*	2.1 (2.0)	2.4 (2.5)*	2.4 (2.2)	2.9 (2.4)*
Conduct problems, mean (SD)	1.5 (1.6)	1.8 (1.6)*	1.5 (1.6)	1.7 (1.6)	1.4 (1.5)	1.9 (1.6)*
Hyperactivity, mean (SD)	3.2 (2.7)	3.1 (2.5)	3.7 (2.8)	3.3 (2.4)	2.8 (2.5)	2.9 (2.5)
Peer relation problems, mean (SD)	1.1 (1.6)	1.8 (1.8)**	1.2 (1.7)	1.8 (1.8)**	1.1 (1.5)	1.8 (1.8)**
Prosocial behaviour ^f^, mean (SD)	8.7 (1.5)	8.8 (1.4)	8.6 (1.6)	8.7 (1.5)	8.8 (1.4)	8.9 (1.3)

*SD* standard deviation

*p values < 0.05; **p < 0.001

^a^ Body mass index (BMI) ≥ 2 standard deviations above the mean according to the 2007 World Health Organization standardized tables

^b^ Measured through the Family Affluence Scale (FAS–II)

^c^ Physical Activity Questionnaire- Children (PAQ–C), scores of 1–5

^d^ Mediterranean – Diet Quality Index (Med–DQI), scores of 1–14

^e^ Strengths and Difficulties Questionnaire by parents; total score (0–40 points). Higher score indicates more problems

^f^ Lower scores indicate lower prosocial behaviour

^†^ Contain missing values

At 9 and 12 years of age, the mean TDS-SDQ scores were 10.5 and 9.3, respectively, in those with obesity, whereas they were 8.7 and 8.0, respectively, in those without obesity (*p* < 0.01). These differences were also apparent when analyses were performed by sex, with higher mean scores in boys than in girls (*p* < 0.05). The scores at 9 years were greater in the group with obesity than in the group without obesity for the scales of emotional symptoms (2.5 vs. 2.1; *p* < 0.001), behavioural problems (2.1 vs. 1.7; *p* < 0.001) and problems with peers (1.8 vs. 1.0; *p* < 0.001). Similar findings were observed at 12 years for the scales of emotional symptoms (2.6 vs. 2.2; *p* < 0.05), behavioural problems (1.8 vs. 1.5; *p* < 0.05) and problems with peers (1.8 vs. 1.1; *p* < 0.001). At both 9 and 12 years of age, these differences were observed in both boys and girls.

The mean TDS-SDQ scores in the groups aged 9 and 12 years, stratified by socioeconomic level, physical activity and quality of diet, are shown in Table S1 of the supplementary material. Children from families with low purchasing power, children with less physical activity, and children with poorer diet quality had higher scores on the TDS-SDQ and on the respective dimensions.

Table 2 shows the results of the analysis of the association of weight status with the SDQ scores. Compared with children without obesity, those who had obesity had higher TDS–SDQ scores, with a β coefficient of 1.12 (95% CI: 0.59; 1.64), as well as more emotional symptoms, behavioural problems and problems with peers, with β coefficients of 0.31 (95% CI: 0.12; 0.51), 0.26 (95% CI: 0.12; 0.41) and 0.57 (95% CI: 0.41; 0.73), respectively. In the analysis stratified by sex, boys showed an increase in the TDS-SDQ score of 0.83 (95% CI: 0.13; 1.53), while in girls the increase in the TDS-SDQ score was 1.42 (95% CI: 0.62; 2.21), although no effect modification was observed (*p* of interaction = 0.325). Similarly, scores on the subscales or dimensions of the SDQ were greater for girls, although no statistically significant effect modification was observed.

**Table 2:** Association between obesity and SDQ scores (0–40 points) in children aged 9 and 12 years

SDQ ^a^	Total ^f^		Boys		Girls		***p-*** interaction ^d^
	β^b^ Coef. ***(95% CI)***	***p-value***	βCoef. ***(95% CI)***	***p-value***	βCoef. ***(95% CI)***	***p-value***	
*Total Difficulties Score (TDS – SDQ)*							
No obesity	(ref)	< 0.001	(ref)		(ref)	< 0.001	0.325
Obesity ^c^	1.12 (0.59; 1.64)		0.83 (0.13; 1.53)	0.020	1.42 (0.62; 2.21)		
*Emotional symptoms*							
No obesity	(ref)	0.002	(ref)	0.079	(ref)	0.009	0.404
Obesity ^c^	0.31 (0.12; 0.51)		0.22 (–0.03; 0.47)		0.42 (0.10; 0.73)		
*Conduct problems*							
*No obesity*	*(ref)*	< 0.001	*(ref)*	0.026	*(ref)*	0.004	0.584
Obesity ^c^	0.26 (0.12; 0.41)		0.22 (0.03; 0.42)		0.31 (0.10; 0.53)		
*Hyperactivity*:							
No obesity	*(ref)*	0.638	*(ref)*		*(ref)*	0.229	0.239
Obesity ^c^	0.05 (–0.17; 0.27)		–0.05 (–0.35; 0.26)	0.765	0.20 (–0.12; 0.52)		
*Peer relation problems*							
No obesity	*(ref)*	< 0.001	*(ref)*		*(ref)*	< 0.001	0.973
Obesity ^c^	0.57 (0.41; 0.73)		0.55 (0.33; 0.76)	< 0.001	0.58 (0.34; 0.81)		
*Prosocial behaviour* ^*e*^							
No obesity	(ref)	0.906	(ref)	0.618	(ref)	0.694	0.373
Obesity ^c^	– 0.01 (–0.13; 0.12)		– 0.05 (–0.23; 0.13)		0.03 (–0.14; 0.21)		

^a^ Strengths and Difficulties Questionnaire by parents: total score (0–40 points). Higher score indicates more problems

^b^ β coefficient estimated by generalized estimating equation linear regression and adjusted for age, household purchasing power, diet quality index (Mediterranean Diet Quality Index) and physical activity (Physical Activity Questionnaire–Children)

^c^ Body mass index (BMI) ≥ 2 standard deviations above the mean according to the 2007 World Health Organization standardized tables

^d^ *p for interaction between obesity and sex*

^*e*^ *Lower scores indicate lower prosocial behaviour*

^f^ *n* = 2473 at age 9 and 2117 at age 12

*95% CI*: 95% confidence interval

Table S2 of the supplementary material presents the results of the sensitivity analysis of the association of weight status with the SDQ scores, excluding the 92 children who were underweight, and similar results were observed. In Table S3, this association was analysed by stratifying the weight status into normal weight, overweight and obesity. Being overweight was only associated with peer relation difficulties and prosocial behaviour subscales.

Table 3 shows the association, in terms of adjusted OR, between obesity and categorized mental health difficulties. Compared with the participants without obesity, those who had obesity had an OR of having problems in the TDS-SDQ of 1.36 (95% CI: 1.09; 1.69), while the ORs were 1.48 (95% CI: 1.21; 1.81) for emotional problems, 1.40 (95% CI: 1.15; 1.70) for conduct problems, and 2.25 (95% CI: 1.83; 2.77) for problems with relationships with peers. The magnitude of the association was also greater in girls than in boys, but this difference did not reach statistical significance when evaluating the interaction.

**Table 3:** Associations between obesity and SDQ scores (normal, borderline/abnormal) in children aged 9 and 12 years

**SDQ** ^**a**^	Total^e^		Boys		Girls		***p*-*interaction*** ^**d**^
	**OR** ^**b**^ ***(95% CI)***	***p-value***	***OR*** ***(95% CI)***	***p-value***	**OR** ***(95% CI)***	***p-value***	
*Total difficulties score (TDS – SDQ)*							
No obesity	1 (ref)	0.005	1 (ref)	0.071	1 (ref)	0.043	0.621
Obesity ^c^	1.36 (1.09; 1.69)		1.30 (0.98; 1.72)		1.42 (1.01; 2.00)		
*Emotional symptoms*							
No obesity	1 (ref)	< 0.001	1 (ref)	0.035	1 (ref)	0.001	0.414
Obesity ^c^	1.48 (1.21; 1.81)		1.35 (1.02; 1.77)		1.63 (1.21; 2.20)		
*Conduct problems*							
No obesity	1 (ref)	0.001	1 (ref)	0.055	1 (ref)	0.004	0.291
Obesity ^c^	1.40 (1.15; 1.70)		1.29 (0.99; 1.66)		1.55 (1.15; 2.09)		
*Hyperactivity*							
No obesity	1 (ref)	0.310	1 (ref)	0.232	1 (ref)	0.765	0.509
Obesity ^c^	0.90 (0.72; 1.11)		0.85 (0.65; 1.11)		0.95 (0.67; 1.34)		
*Peer relation problems*							
No obesity	1 (ref)	< 0.001	1 (ref)	< 0.001	1 (ref)	< 0.001	0.839
Obesity ^c^	2.25 (1.83; 2.77)		2.18 (1.65; 2.88)		2.33 (1.69; 3.20)		
*Prosocial behaviour*							
No obesity	1 (ref)	0.594	1 (ref)	0.763	1 (ref)	0.220	0.176
Obesity ^c^	0.87 (0.52; 1.45)		1.09 (0.61; 1.94		0.47 (0.14; 1.57)		

^a^ Strengths and Difficulties Questionnaire by parents: Total Difficulties Score (TDS – SDQ), borderline/abnormal: ≥14 points; and the borderline/abnormal subscales: emotional problems (≥ 4 points), conduct problems (≥ 3 points), hyperactivity (≥ 6 points), peer relation problems (≥ 3 points). Strengths: Prosocial behaviour (≤ 6 points)

^b^ Odds ratios (ORs) estimated by GEE logistic regression and adjusted for age, household purchasing power, diet quality index (Mediterranean Diet Quality Index) and physical activity (Physical Activity Questionnaire–Children)

^c^ Obesity: body mass index (BMI) ≥ 2 standard deviations above the mean according to the 2007 World Health Organization standardized tables

^d^ *p* for - interaction between obesity and sex

^e^ *n* = 2473 at age 9 and 2117 at age 12

95% CI: 95% confidence interval

Finally, the association between obesity and mental health did not change between ages 9 and 12 for most estimates. Only one statistically significant interaction was observed with the hyperactivity dimension, with the β coefficient decreasing from 0.19 at age 9 to −0.18 at age 12 (p for interaction = 0.028) (data not shown).

## Discussion

In this study, the association between obesity and mental health problems was evaluated in a sample of 9- and 12-year-old Spanish children. The results show an association of childhood obesity with mental health difficulties, especially with emotional symptoms, behavioural problems and relationship problems with peers.

Nujic et al. [13] performed a systematic review, and in a subanalysis limited to 10 cross-sectional studies in which the SDQ was used, the authors reported an OR of 1.36, similar to the estimate in our study. Subsequent studies have also revealed a positive association between obesity and the risk of mental health difficulties. This is the case for the study by Beynon et al. in the UK [34], which involved a sample of 11,279 participants aged 4–15 years from the Wales Health Survey. The authors found an association of small, statistically significant magnitude (OR: 1.02) of obesity with TDS-SDQ scores ≥ 20. Similarly, in a study of 871 11-year-olds in New Zealand [35], those with a BMI classifying them as overweight or obese had a greater risk of mental health problems as determined by the TDS-SDQ, with an estimated OR of 2.32.

Regarding the association of weight status with scores on the different dimensions of the SDQ, the behavioural, emotional and relationship problems found in our study are consistent with what was observed in other studies [36–38]. A recent cross-sectional study [36] carried out in Germany with two age groups, 4–10 and 11–18 years, found a positive association between being overweight and problems with peers in the group aged 4–10 years and in the group aged 11–18 years and between being overweight and emotional symptoms in the 11–18 year-old group. A study in England in children aged 5–16 years [39] also showed a positive association between obesity and emotional problems. Similarly, a cross-sectional study conducted in Norway [18] of 12–13-year-old children revealed an association between excess weight and problems with peers, with an estimated OR of 2.06. This relationship can be explained by the fact that at these ages, children and adolescents with obesity suffer more social rejection, isolation, and harassment and are more exposed to social stigmatization than normal-weight children are [17–19]. Being overweight in children and adolescents can be a marker of social marginalization and the reason why they have fewer friends due to victimization by peers [40, 41]. In addition, these children (overweight, marginalized, victimized, etc.) frequently present psychological problems and psychiatric disorders [42]. It has been suggested that problems with peers could be an important underlying factor for the development of mental health problems in children, especially in adolescents [43, 44].

Concerning hyperactivity, the null results found in our study are consistent with those of other authors who used the same instrument (SDQ) to assess mental health in the context of weight status in children [36, 37].

A relevant aspect of the study was to evaluate whether the association of weight status with mental health difficulties was modified according to sex, an aspect rarely addressed in the literature. An association of greater magnitude could be expected in girls because they are more sensitive to the experiences of stigmatization due to their weight status than boys are, and this increased sensitivity gives rise to more frequent mental health problems, anxiety and psychological disorders [43]. In our analysis, the association between weight status and mental health problems was in the same direction for both boys and girls but was more intense for girls, although without being significant. However, in the systematic review by Nujic et al. [13] an OR of 2 was observed in the studies focused on boys (4 studies), while no association was observed in the other 4 studies focused on girls. Subsequent studies with sufficient statistical power should add more evidence on the existence of a modification of the effect on this association according to sex.

These results have important implications for public health, clinical practice and future research. Mental health is possibly one of the least valued areas in the approach to childhood obesity in health services. Therefore, public health policies, in addition to promoting healthy habits of diet and physical activity, should include an evaluation of the mental health of children with obesity, considering the prejudices in social relationships and the concerns about weight and body image that arise at an early age. Therefore, it is necessary to promote the sensitivity and empathy of parents, caregivers, friends, and health personnel to identify mental health difficulties in overweight children and adolescents.

### Limitations and strengths

For a correct interpretation of the results, some limitations and strengths should be noted. First, the observational design of the study does not allow for the estimation of causal associations between obesity and mental health difficulties. The directionality of the association between obesity and mental health problems remains unclear. It is not yet clear whether psychiatric disorders and psychological problems are a cause or a consequence of childhood obesity or whether there are common factors that promote both obesity and psychiatric disorders in susceptible children [45]. Another possible limitation is that children’s mental health difficulties were reported only by parents, which may result in discrepancies between parents’ reports and the children’s self-reports. These differences could provide complementary insights for the assessment of mental health risk in children and adolescents [46, 47].

Large prospective studies are necessary to establish a temporal relationship between these variables. On the other hand, although our models included adjustments for the main confounding variables, some residual confounding cannot be ruled out.

Regarding the strengths of this study, it should be noted that this is one of the few population studies that investigates mental health problems in children and obesity in Spain using the SDQ. In addition, this study on obesity was carried out with a specific longitudinal design in a representative sample of the child population. On the other hand, anthropometric measurements are objective criteria and were all performed in a standardized way; therefore, these measurements are subject to fewer validity errors than those reported by parents or children [48]. Finally, the main confounding variables, including diet and physical activity, were considered.

## Conclusions

The results of this study show that children living with obesity are more likely to experience difficulties and mental health problems. This relationship highlights the importance of incorporating screening for mental health problems and psychological support in public health strategies for the prevention and control of childhood obesity.

## Supplementary Information

Supplementary Material 1.

Supplementary Material 2.

Supplementary Material 3.

## Data Availability

Available from the corresponding author upon request.

## Abbreviations

BMI: Body Mass Index
CI: Confidence Interval
ELOIN: Longitudinal Childhood Obesity Study
GEE: Generalized Estimating Equation
OR: Odds Ratio
SD: Standard Deviation
SDQ: Strengths and Difficulties Questionnaire
WHO: World Health Organization

## References

[1] Horesh A, Tsur AM, Bardugo A, Twig G. Adolescent and childhood obesity and excess morbidity and mortality in young adulthood-a systematic review. Curr Obes Rep. 2021;10(3):301–10. 10.1007/s13679-021-00439-9. 33950400 10.1007/s13679-021-00439-9

[2] García-Solano M et al. oct., «Weight status in the 6- to 9-year-old school population in Spain: results of the ALADINO 2019 Study», *Nutr Hosp*, vol. 38, n.^o^ 5, pp. 943–953, 2021, 10.20960/nh.03618 10.20960/nh.03618 34304573

[3] World Health Organizationn. «Mental health of adolescents». [En línea]. Disponible en: https://www.who.int/news-room/fact-sheets/detail/adolescent-mental-health

[4] Barican JL, Yung D, Schwartz C, Zheng Y, Georgiades K, Waddell C. Prevalence of childhood mental disorders in high-income countries: a systematic review and meta-analysis to inform policymaking. Evid Based Ment Health. 2022;25(1):36–44. 10.1136/ebmental-2021-300277. 34281985 10.1136/ebmental-2021-300277 PMC8788041

[5] Simmonds M, Llewellyn A, Owen CG, Woolacott N. Predicting adult obesity from childhood obesity: a systematic review and meta-analysis. Obes Rev. 2016;17(2):95–107. 10.1111/obr.12334. 26696565 10.1111/obr.12334

[6] Pulgarón ER. Childhood obesity: a review of increased risk for physical and psychological comorbidities. Clin Ther. 2013;35(1):A18-32. 10.1016/j.clinthera.2012.12.014. 23328273 10.1016/j.clinthera.2012.12.014 PMC3645868

[7] Sanders RH, Han A, Baker JS, Cobley S. Childhood obesity and its physical and psychological co-morbidities: a systematic review of Australian children and adolescents. Eur J Pediatr. 2015;174(6):715–46. 10.1007/s00431-015-2551-3. 25922141 10.1007/s00431-015-2551-3

[8] Sutaria S, Devakumar D, Yasuda SS, Das S, Saxena S. Is obesity associated with depression in children? Systematic review and meta-analysis. Arch Dis Child. 2019;104(1):64–74. 10.1136/archdischild-2017-314608. 29959128 10.1136/archdischild-2017-314608

[9] Schwimmer JB, Burwinkle TM, Varni JW. Health-related quality of life of severely obese children and adolescents. JAMA. 2003;289(14):1813–9. 10.1001/jama.289.14.1813. 12684360 10.1001/jama.289.14.1813

[10] Ottova V, Erhart M, Rajmil L, Dettenborn-Betz L, Ravens-Sieberer U. Overweight and its impact on the health-related quality of life in children and adolescents: results from the European KIDSCREEN survey. Qual Life Res. 2012;21(1):59–69. 10.1007/s11136-011-9922-7. 21557001 10.1007/s11136-011-9922-7

[11] Rankin J, et al. Psychological consequences of childhood obesity: psychiatric comorbidity and prevention. Adolesc Health Med Ther. 2016;7:125–46. 10.2147/AHMT.S101631. 27881930 10.2147/AHMT.S101631 PMC5115694

[12] Kokka I, Mourikis I, Bacopoulou F. Psychiatric disorders and obesity in childhood and adolescence-a systematic review of cross-sectional studies. Children. 2023;10(2):285. 10.3390/children10020285. 36832413 10.3390/children10020285 PMC9955505

[13] Nujić D, Musić Milanović S, Milas V, Miškulin I, Ivić V, Milas J. Association between child/adolescent overweight/obesity and conduct disorder: a systematic review and meta-analysis. Pediatr Obes. 2021;16(5):e12742. 10.1111/ijpo.12742. 33348469 10.1111/ijpo.12742

[14] Eiffener E, et al. «The influence of preschoolers’ emotional and behavioural problems on obesity treatment outcomes: secondary findings from a randomized controlled trial». Pediatr Obes. nov. 2019;14:e12556. 10.1111/ijpo.12556. n.^o^ 11. 10.1111/ijpo.12556 31290278

[15] Turco G, Bobbio T, Reimão R, Rossini S, Pereira H, Barros Filho A. Quality of life and sleep in obese adolescents. Arq Neuropsiquiatr. 2013;71(2):78–82. 10.1590/s0004-282x2013005000008. 23306207 10.1590/s0004-282x2013005000008

[16] Palmer AJ, et al. Childhood overweight and obesity and back pain risk: a cohort study of 466 997 children. BMJ Open. 2020;10(9):e036023. 10.1136/bmjopen-2019-036023. 32948552 10.1136/bmjopen-2019-036023 PMC7500301

[17] Schreckenbach J, Reis O, Häßler F. [Overweight/Obesity of Children and Adolescents and its Association with Internalising and Externalising Disorders]. Prax Kinderpsychol Kinderpsychiatr. 2021;70(3):182–97. 10.13109/prkk.2021.70.3.182. 33641646 10.13109/prkk.2021.70.3.182

[18] Hestetun I, Svendsen MV, Oellingrath IM. Associations between overweight, peer problems, and mental health in 12-13-year-old Norwegian children. Eur Child Adolesc Psychiatry. 2015;24(3):319–26. 10.1007/s00787-014-0581-4. 25012463 10.1007/s00787-014-0581-4

[19] Yu B. Kindergarten obesity and academic achievement: the mediating role of weight bias. Front Psychol. 2021;12:640474. 10.3389/fpsyg.2021.640474. 33935891 10.3389/fpsyg.2021.640474 PMC8086407

[20] Patalay P, Hardman CA. Comorbidity, codevelopment, and temporal associations between body mass index and internalizing symptoms from early childhood to adolescence. JAMA Psychiatry. 2019;76(7):721–9. 10.1001/jamapsychiatry.2019.0169. 30892586 10.1001/jamapsychiatry.2019.0169 PMC6583661

[21] Newson L, Abayomi J. Reframing interventions for optimal child nutrition and childhood obesity: the importance of considering psychological factors. Proc Nutr Soc. 2024;(1):12. 10.1017/S0029665124000028. 10.1017/S0029665124000028 38205619

[22] Ortiz-Marrón H, Cuadrado-Gamarra JI, Esteban-Vasallo M, Cortés-Rico O, Sánchez-Díaz J, Galán-Labaca I. The longitudinal childhood obesity study (ELOIN): design, participation and characteristics of the baseline sample. Revista Española de Cardiología (English Edition). 2016;69(5):521–3. 10.1016/j.rec.2016.01.017. 10.1016/j.rec.2016.01.017 27005760

[23] Pérez-Farinós N, Galán I, Ordobás M, Zorrilla B, Cantero JL, Ramírez R. A sampling design for a sentinel general practitioner network. Gac Sanit. 2009;23(3):186–91. 10.1016/j.gaceta.2008.05.010. 19269065 10.1016/j.gaceta.2008.05.010

[24] WHO, «World Health Organizatio. (2006). Child Growth Standards. Geneva. Switzerland.», WHO. [En línea]. Disponible en: https://www.who.int/childgrowth/standards/en/

[25] Goodman R. Psychometric properties of the strengths and difficulties questionnaire. J Am Acad Child Adolesc Psychiatry. 2001;40(11):1337–45. 10.1097/00004583-200111000-00015. 11699809 10.1097/00004583-200111000-00015

[26] Stone LL, Otten R, Engels RCME, Vermulst AA, Janssens JMAM. Psychometric properties of the parent and teacher versions of the strengths and difficulties questionnaire for 4- to 12-year-olds: a review. Clin Child Fam Psychol Rev. 2010;13(3):254–74. 10.1007/s10567-010-0071-2. 20589428 10.1007/s10567-010-0071-2 PMC2919684

[27] Ortuño-Sierra J, Aritio-Solana R, Fonseca-Pedrero E. Mental health difficulties in children and adolescents: the study of the SDQ in the Spanish National Health Survey 2011–2012. Psychiatry Res. 2018;259:236–42. 10.1016/j.psychres.2017.10.025. 29091822 10.1016/j.psychres.2017.10.025

[28] Goodman R. The strengths and difficulties questionnaire: a research note. J Child Psychol Psychiatry. 1997;38(5):581–6. 10.1111/j.1469-7610.1997.tb01545.x. 9255702 10.1111/j.1469-7610.1997.tb01545.x

[29] Theunissen MHC, de Wolff MS, Eekhout I, Mieloo CL, Stone LL, Reijneveld SA. The strengths and difficulties questionnaire parent form: Dutch norms and validity. BMC Pediatr. 2022;22(1):202. 10.1186/s12887-022-03274-6. 35413892 10.1186/s12887-022-03274-6 PMC9004049

[30] Torsheim T, et al. Psychometric validation of the revised family affluence scale: a latent variable approach. Child Indic Res. 2016;9:771–84. 10.1007/s12187-015-9339-x. 27489572 10.1007/s12187-015-9339-x PMC4958120

[31] Gerber M. Qualitative methods to evaluate Mediterranean diet in adults. Public Health Nutr. 2006;9(1A):147–51. 10.1079/phn2005937. 16512962 10.1079/phn2005937

[32] Manchola-Gonzalez J et al. «Validation of the PAQ-C questionnaire to assess physical activity in Spanish older children», *Physiotherapy (United Kingdom)* 2015, 101 SPPL. Accedido: 9 de mayo de 2017. [En línea]. Disponible en: https://www.embase.com/#advancedSearch/resultspage/history.1/page.1/25.items/orderby.date/source

[33] Fajardo-Bullón F, Rasskin-Gutman I, León-Del Barco B, Ribeiro Dos Santos EJ, Iglesias Gallego D. International and Spanish findings in scientific literature about minors’ mental health: predictive factors using the strengths and difficulties questionnaire. Int J Environ Res Public Health. 2019;16(9):1603. 10.3390/ijerph16091603. 31071907 10.3390/ijerph16091603 PMC6539595

[34] Beynon C. Association between children living with obesity and mental health problems: a data analysis of the Welsh Health Survey, UK. BMC Public Health. 2023;23(1):383. 10.1186/s12889-023-15293-8. 36823604 10.1186/s12889-023-15293-8 PMC9947886

[35] Slykerman RF, et al. Physical activity, sleep, body mass index, and associated risk of behavioral and emotional problems in childhood. J Dev Behav Pediatr. 2020;41(3):187–94. 10.1097/DBP.0000000000000754. 32004246 10.1097/DBP.0000000000000754

[36] Förster L-J, et al. Mental health in children and adolescents with overweight or obesity. BMC Public Health. 2023;23(1):135. 10.1186/s12889-023-15032-z. 36658514 10.1186/s12889-023-15032-z PMC9849834

[37] Pitrou I, Shojaei T, Wazana A, Gilbert F, Kovess-Masféty V. Child overweight, associated psychopathology, and social functioning: a French school-based survey in 6- to 11-year-old children. Obesity. 2010;18(4):809–17. 10.1038/oby.2009.278. 19713951 10.1038/oby.2009.278

[38] Halfon N, Larson K, Slusser W. Associations between obesity and comorbid mental health, developmental, and physical health conditions in a nationally representative sample of US children aged 10 to 17. Acad Pediatr. 2013;13(1):6–13. 10.1016/j.acap.2012.10.007. 23200634 10.1016/j.acap.2012.10.007

[39] Tiffin PA, Arnott B, Moore HJ, Summerbell CD. Modelling the relationship between obesity and mental health in children and adolescents: findings from the Health Survey for England 2007. Child Adolesc Psychiatry Ment Health. 2011;5:31. 10.1186/1753-2000-5-31. 21982578 10.1186/1753-2000-5-31 PMC3213165

[40] Strauss RS, Pollack HA. Social marginalization of overweight children. Arch Pediatr Adolesc Med. 2003;157(8):746–52. 10.1001/archpedi.157.8.746. 12912779 10.1001/archpedi.157.8.746

[41] Puhl RM, Lessard LM. Weight Stigma in Youth: prevalence, consequences, and considerations for clinical practice. Curr Obes Rep. 2020. 10.1007/s13679-020-00408-8. 33079337 10.1007/s13679-020-00408-8

[42] Hay DF, Payne A, Chadwick A. Peer relations in childhood. J Child Psychol Psychiatry. 2004;45(1):84–108. 10.1046/j.0021-9630.2003.00308.x. 14959804 10.1046/j.0021-9630.2003.00308.x

[43] Russell-Mayhew S, McVey G, Bardick A, Ireland A. Mental health, wellness, and childhood overweight/obesity. J Obes. 2012;2012:281801. 10.1155/2012/281801. 22778915 10.1155/2012/281801 PMC3388583

[44] Lessard LM, Puhl RM. Weight-based cybervictimization: implications for adolescent health. Pediatr Obes. 2022;17(6):e12888. 10.1111/ijpo.12888. 35076170 10.1111/ijpo.12888

[45] Sagar R, Gupta T. Psychological Aspects of Obesity in Children and Adolescents. Indian J Pediatr. 2018;85(7):554–9. 10.1007/s12098-017-2539-2. 29150753 10.1007/s12098-017-2539-2

[46] Bajeux E, Klemanski D, Husky M, et al. Factors associated with parent–child discrepancies in reports of mental health disorders in young children. Child Psychiatry Hum Dev. 2018;49:1003–10. 10.1007/s10578-018-0815-7. 29869765 10.1007/s10578-018-0815-7

[47] Van Roy B, Groholt B, Heyerdahl S, et al. Understanding discrepancies in parent-child reporting of emotional and behavioural problems: effects of relational and socio-demographic factors. BMC Psychiatry. 2010;10:56. 10.1186/1471-244X-10-56. 20637090 10.1186/1471-244X-10-56 PMC2912799

[48] Esteban-Vasallo MD, et al. Accuracy of anthropometric measurements and weight status perceptions reported by parents of 4-year-old children. Public Health Nutr. 2019. 10.1017/S1368980019003008. 31685044 10.1017/S1368980019003008 PMC10200599

