# Supplementary material for: Association between obesity and mental health problems among Spanish children aged 9 and 12 years: the ELOIN study
Source: BMC Public Health. 2026 Feb 7;26:857. doi: 10.1186/s12889-026-26349-w (PMC12977668; doi:10.1186/s12889-026-26349-w)
Supplement: Supplementary file 1 — Supplementary Material 1. [file 12889_2026_26349_MOESM1_ESM.pdf]

SUPPLEMENTARY INFORMATION

Table S1. SDQ scores according to covariates in children at 9 and 12 years of age

	9 years			12 years		
	**Total (n = 2473)**	**Boys (n = 1207)**	**Girls (n = 1266)**	**Total (n = 2119)**	**Boys (n = 1025)**	**Girls (n = 1094)**
Total Difficulties Score [a] (TDS – SDQ), mean (SD)	8.9 (5.4)	9.3 (5.4)	8.6 (5.2)**	8.19 (5.6)	8.57 (5,7)	7.84 (5.5)*
Household purchasing power [b] (%)						
Low	10.8 (5.7)**	10.8 (5.4)**	10.8 (6.0)**	9.7 (6.2)**	9.4 (6.1)*	10.0 (6.2)**
Medium	9.8 (5.3)	10.3 (5.4)	9.4 (5.2)	8.5 (5.5)	9.1 (5.5)	7.9 (5.5)
High	8.2 (5.0)	8.6 (5.2)	7.8 (4.8)	7.7 (5.5)	8.1 (5.7)	7.4 (5.2)
Physical activity [c], mean (SD)						
Low	11.5 (6.6)**	13.7 (7.51)**	10.2 (5.6)*	8.9 (6.1)*	9.8 (4.5)	8.3 (5.8)*
Medium	9.0 (5.3)	9.5 (5.4)	8.5 (5.1)	7.8 (5.4)	8.3 (5.5)	7.2 (5.2)
High	8.3 (5.2)	8.4 (5.0)	8.3 (5.4)	8.5 (5.9)	8.5 (6.1)	8.6 (5.8)
Mediterranean – Diet quality score [d] mean (SD)						
Poor (10–14)	9.2 (6.1)*	9.7 (6.4)	8.5 (5.6)*	9.3 (5. 8)*	9.0 (5.8)	9.7 (5.8)*
Medium–poor (8–9)	9.7 (5.2)	9.9 (5.3)	9.5 (5.1)	8.6 (5.6)	8.8 (5.7)	8.4 (5.4)
Good/medium good (1-7)	8.7 (5.3)	9.1 (5.4)	8.4 (5.2)	7.9 (5.6)	8.4 (5.7)	7.4 (5.4)

SD standard deviation
* *p* value < 0.05; ***p* value < 0.001
[a] Strengths and Difficulties Questionnaire by parents: Total Difficulties Score (0–40 points)
[b] Measured through the Family Affluence Scale (FAS–II)
[c] Physical Activity Questionnaire–Children (PAQ–C), score of 1–5
[d] Mediterranean–Diet Quality Index (Med–DQI), score of 1–14
