# Supplementary material for: Association between obesity and mental health problems among Spanish children aged 9 and 12 years: the ELOIN study
Source: BMC Public Health. 2026 Feb 7;26:857. doi: 10.1186/s12889-026-26349-w (PMC12977668; doi:10.1186/s12889-026-26349-w)
Supplement: Supplementary file 2 — Supplementary Material 2. [file 12889_2026_26349_MOESM2_ESM.pdf]

SUPPLEMENTARY INFORMATION

Table S2. Association between obesity and SDQ score (0-40 points) in children at 9 and 12 years of age, excluding underweight children (zBMI ≤2 standard deviations below the mean)

	Total		Boys		Girls		
**SDQ** [a]	**β** [b] **Coef. *(95% CI)***	***p-value***	**β Coef. *(95% CI)***	***p-value***	**β Coef. *(95% CI)***	***p-value***	***p-*interaction** [d]
Total Difficulties Score (TDS – SDQ)							
No obesity	(ref)	<0.001	(ref)		(ref)	0.001	0.348
Obesity [c]	1.09 (0.57; 1.62)		0.82 (0.12; 1.52)	0.022	1.39 (0.59; 2.18)		
Emotional symptoms							
No obesity	(ref)	0.002	(ref)	0.094	(ref)	0.010	0.412
Obesity [c]	0.30 (0.11; 0.50)		0.21 (–0.04; 0.46)		0.41 (0.10; 0.72)		
Conduct problems							
No obesity	*(ref)*	<0.001	*(ref)*	0.026	*(ref)*	0.004	0.598
Obesity [c]	0.26 (0.12; 0.41)		0.22 (0.03; 0.42)		0.31 (0.10; 0.52)		
Hyperactivity:							
No obesity	*(ref)*	0.638	*(ref)*		*(ref)*	0.226	0.275
Obesity [c]	0.05 (–0.17; 0.27)		–0.04 (–0.35; 0.26)	0.765	0.19 (–0.14; 0.50)		
Peer relation problems							
No obesity	*(ref)*	<0.001	*(ref)*		*(ref)*	<0.001	0.956
Obesity [c]	0.54 (0.32; 0.75)		0.57 (0.34; 0.81)	<0.001	0.56 (0.34; 0.78)		
Prosocial behaviour [e]							
No obesity	(ref)	0.906	(ref)	0.618	(ref)	0.694	0.375
Obesity [c]	–0.01 (–0.13; 0.12)		– 0.05 (–0.23; 0.13)		0.03 (–0.14; 0.21)		

[a] Strengths and Difficulties Questionnaire by parents: Total Difficulties Score (0–40 points). Higher score indicates more problems
[b] β coefficient estimated by GEE linear regression and adjusted for age, household purchasing power, diet quality index (Mediterranean Diet Quality Index) and physical activity (Physical Activity Questionnaire-Children)
[c] Obesity: body mass index (BMI) ≥2 standard deviations above the mean according to the 2007 World Health Organization standardized tables
[d] *p for interaction between obesity and sex*
[e] *Lower scores indicate lower prosocial behaviour*
95% CI: 95% confidence interval
