# Supplementary material for: Association between obesity and mental health problems among Spanish children aged 9 and 12 years: the ELOIN study
Source: BMC Public Health. 2026 Feb 7;26:857. doi: 10.1186/s12889-026-26349-w (PMC12977668; doi:10.1186/s12889-026-26349-w)
Supplement: Supplementary file 3 — Supplementary Material 3. [file 12889_2026_26349_MOESM3_ESM.pdf]

SUPPLEMENTARY INFORMATION

Table S3. Association between weight status (normal weight, overweight, and obesity) and SDQ score (0-40 points) in children at 9 and 12 years of age

SDQ [a]	Total		Boys		Girls		
	β [b] Coef. *(95% CI)*	*p-value*	β Coef. *(95% CI)*	*p-value*	β Coef. *(95% CI)*	*p-value*	p-interaction [f]
Total Difficulties Score (TDS – SDQ)							
Normal weight [c]	(ref)		(ref)		(ref)		
Overweight [d]	–0.04 (–0.40; 0.32)	0809	–0.14 (–0.67; 0.39)	0.609	0.11 (–0.39; 0.60)	0.678	0.498
Obesity [e]	1.07 (0.53; 1.62	<0.001	0.76 (0.03; 1.49)	0.041	1.43 (0.61; 2.25	0.001	0.275
Emotional symptoms							
Normal weight [c]	*(ref)*		*(ref)*	0.079	*(ref)*		
Overweight [d]	–0.00 (–0.14; 0.14)	0.996)	–0.04 (–0.23; 0.16)	0.713	0.06 (–0.15; 0.27)	0.605	0.579
Obesity [e]	0.30 (0.10; 0.50)	0.003	0.20 (–0.06; 0.46)	0.130	0.43 (0.11; 0.75)	0.009	0.109
Conduct problems							
Normal weight [c]	(ref)		(ref)		(ref)		
Overweight [d]	–0.06 (–0.16; 0.04)	0.263	–0.15 8–0.30; 0.00)	0.053	0.04 (–0.11; 0.19)	0.611	0.077
Obesity [e]	0.24 (0.09; 0.39)	0.002	0.16 (–0.04; 0.37)	0.120	0.32 (0.11; 0.54)	0.003	0.326
Hyperactivity:							
Normal weight [c]	(ref)		(ref)		(ref)		
Overweight [d]	–0.12 (–0.29; 0.06)	0.184	–0.10 (–0.37; 0.16)	0.443	–0.12 (–035; 0.11)	0.303	0.892
Obesity [e]	–0.01 (–0.24; 0.23)	0.962	–0.09 (–0.42; 0.24)	0.593	0.13 (–0.21; 0.46	0.462	0.332
Peer relation problems							
Normal weight [c]	(ref)		(ref)		(ref)		
Overweight [d]	0.14 (0.04; 0.24)	0.006	0.13 (–0.02; 0.29)	0.085	0.16 (0.02; 0.30)	0.025	0.913
Obesity [e]	0.62 (0.45; 0.78)	<0.001	0.59 (0.37; 0.81)	<0.001	0.63 (0.39; 0.87)	<0.001	0.937
Prosocial behaviour [g]							
Normal weight [c]	(ref)		(ref)		(ref)		
Overweight [d]	0.12 (0.02; 0.22)	0.020	0.18 (0.03; 0.33)	0.019	0.05 (–0.08; 0.18)	0.449	0.138
Obesity [e]	0.03 (–0.10; 0.17)	0.613	0.02 (–0.18; 0.21)	0.871	0.05 (–013; 0.23)	0.587	0.651

[a] Strengths and Difficulties Questionnaire by parents: Total Difficulties Score (0–40 points). Higher score indicates more problems
[b] β coefficient estimated by generalized estimating equation linear regression and adjusted for age, household purchasing power, diet quality index (Mediterranean Diet Quality Index) and physical activity (Physical Activity Questionnaire-Children)
[c] Normal weight: body mass index (BMI) ≥2 standard deviations (SD) and ≤1 SD above the mean according to the 2007 World Health Organization (WHO) standardized tables
[d] Overweight: BMI ≥1 SD and ≤2 SD above the mean according to the 2007 WHO standardized tables
[e] Obesity: BMI ≥2 SD above the mean according to the 2007 WHO standardized tables
[f] *p for interaction between overweight, obesity, and sex*
[g] *Lower scores indicate lower prosocial behaviour*
95% CI: 95% confidence interval
